# Supplementary material for: Differences in treatment and outcome of patients with ST- elevation myocardial infarction (STEMI) and non-STEMI in Germany
Source: PLoS One. 2026 Oct 15;21(10):e0343889. doi: 10.1371/journal.pone.0343889 (PMC13671558; doi:10.1371/journal.pone.0343889)
Supplement: Supplementary file 1 [file pone.0343889.s001.pdf]

S1 Table.

ICD-10-GM Codes/ OPS diagnosis and procedure codes as well as ATC codes relevant for analyses.

**Parameter**	**Codes**
**Acute myocardial infarction**<br>**STEMI**<br>**NSTEMI**	 ICD: I21.0, I21.1; I21.2, I22.0, I22.1, I22.8<br>ICD: I21.3, I21.4, I21.9, I22.9
**Previous myocardial infarction**	ICD: I21.-; I22.-
**No. of diseased coronary vessels**<br>**1**<br>**2**<br>**3**	ICD:<br>I25.11<br>I25.12<br>I25.13
**Peripheral artery disease (PAD)**<br>**PAD 1-3**<br>**PAD 4-6**	ICD:<br>I70.20, I70.21, I70.22 (from 2015)<br>I70.22 (until 2014), I70.23 I70.24 I70.25(from 2015)
**Congestive heart failure (CHF)**<br>**NYHA I**<br>**NYHA II**<br>**NYHA III**<br>**NYHA IV**	ICD:<br>I50.11<br>I50.12<br>I50.13<br>I50.14
**Right ventricular heart failure**	ICD: I50.0
**Previous cerebrovascular disease**	ICD: I65, I66, I67.2
**Previous stroke**	ICD: I63.-; I64.-; I69.3; I69.4
**Atrial fibrillation**	ICD: I48
**Chronic kidney disease**	ICD: N18, N19
**Diabetes mellitus non-specified**	ICD: E10-E14
**Dyslipidemia**	ICD: E78
**Cancer**	ICD: C
**Psychiatric disorder**	ICD: F13, F33, F41, F50, F51, X59
**Obesity**	ICD: E66
**Nicotine abuse**	ICD: F17
**Arterial hypertension**	ICD: I10-I15
**Cerebral insult**	ICD: I63, I64,I69.3, I69.4
**Coronary artery bypass grafting**	ICD: Z95.1, OPS: 5-36
**Percutaneous coronary intervention**	OPS: 8-837.0
**Diagnostic cardiac catheterization**	OPS: 1-275
**Drug eluting stent (DES) implantation**	OPS: 8-837.m
**Bare metal stent (BMS) implantation**	OPS: 8-837.k
**Circulatory support device (Impella®)**	OPS: 8-83a.3
**Intra-aortic balloon pump**	OPS: 8-83a.0
**Extra corporal membrane oxygenation**	OPS: 8-851, 8-852
**Use of GPIIb/IIIa inhibitors**	OPS: 6-002.j, 6-002.k, 6-002.n
**Sepsis**	ICD: A41
**Acute renal failure**	ICD: N17
**Renal replacement therapy**	OPS: 8-853, 8-854, 8-855
**Shock**	ICD: T81.1, R57.0
**Cardiogenic shock**	ICD: T81.1, R57.0<br>OPS: 8-852, 8-83a.0, 8-83a.3, 8-771
**Cardiopulmonary resuscitation**	OPS: 8-771
**Blood transfusion**	OPS: 8-800.c
**Bleeding**	ICD: K92, H44.8, T81.0, T81.2, T81.3, T81.7
**Valve replacement**	ICD: Z95.2, OPS: 5-351, 5-352, 5-353, 5-354; 5-535a
**Oral anticoagulants**	ATC: B01AA, B01AE07, B01AF (from 2013), B01AX06 (only 2012), B01AX08 (only 2012)

**Platelet aggregation inhibitors (PAI)**	ATC: B01AC
**Statins**	ATC: C10AA, C10BA, C10BX
**Beta blockers**	ATC: C07
**ACE- inhibitors/ Angiotensin II receptor blockers (ACEIs/ARBs)**	ATC: C09A-D

Abbreviation: Angiotensin converting enzyme, ACE; Anatomical therapeutic chemical, ATC; international classification of disease-10-German modification, ICD-10-GM Code; non-ST-elevation myocardial infarction, NSTEMI; New York heart association, NYHA; operation and procedure code, OPS; ST-elevation myocardial infarction, STEMI
