# Supplementary material for: Differences in treatment and outcome of patients with ST- elevation myocardial infarction (STEMI) and non-STEMI in Germany
Source: PLoS One. 2026 Oct 15;21(10):e0343889. doi: 10.1371/journal.pone.0343889 (PMC13671558; doi:10.1371/journal.pone.0343889)
Supplement: Supplementary file 2 [file pone.0343889.s002.pdf]

S2 Table. Event rate including overall death, re-infarction or death and MACCE: Event rates were given by 1- survival rates determined by Kaplan Meier estimate.

	Entire AMI cohort	STEMI	NSTEMI	P value
**Overall death – % (95% CI)**				<0.001
1 year rate	23.2% (23.1 – 23.3%)	21.5% (21.4 – 21.7%)	24.0% (23.9 – 24.1%)	
2 years rate	29.0% (28.9 – 29.1%)	25.0% (24.8 – 25.2%)	31.0% (30.8 – 31.1%)	
3 years rate	34.1% (34.0 – 34.2%)	28.2% (28.0 – 28.4%)	37.0% (36.9 – 37.2%)	
4 years rate	38.9% (38.8 – 39.0%)	31.4% (31.2 – 31.7%)	42.6% (42.4 – 42.8%)	
5 years rate	43.4% (43.3 – 43.5%)	34.7% (34.5 – 35.0%)	47.7% (47.6 – 47.9%)	
6 years rate	47.6% (47.5 – 47.8%)	38.0% (37.8 – 38.3%)	52.5% (52.3 – 52.7%)	
7 years rate	51.6% (51.5 – 51.8%)	41.5% (41.2 – 41.8%)	56.7% (56.5 – 57.0%)	
8 years rate	55.4% (55.2 – 55.6%)	44.9% (44.6 – 45.2%)	60.8% (60.5 – 61.0%)	
9 years rate	59.0% (58.8 – 59.2%)	48.2% (47.9 – 48.6%)	64.5% (64.2 – 64.7%)	
10 yrs rate	62.3% (62.0 – 62.7%)	51.8% (51.2 – 52.4%)	67.7% (67.3 – 68.2%)	
**Reinfarction or death – % (95% CI)**				<0.001
1 year rate	31.5% (31.3 – 31.6%)	28.9% (28.7 – 29.1%)	32.7% (32.6 – 32.9%)	
2 years rate	38.5% (38.3 – 38.6%)	33.6% (33.4 – 33.8%)	40.9% (40.7 – 41.0%)	
3 years rate	44.0% (43.9 – 44.2%)	37.5% (37.3 – 37.8%)	47.2% (47.1 – 47.4%)	
4 years rate	49.0% (48.8 – 49.1%)	41.3% (41.1 – 41.5%)	52.8% (52.6 – 53.0%)	
5 years rate	53.4% (53.3 – 53.6%)	44.9% (44.7 – 45.2%)	57.7% (57.5 – 57.9%)	
6 years rate	57.5% (57.4 – 57.7%)	48.5% (48.3 – 48.8%)	62.1% (61.9 – 62.2%)	
7 years rate	61.3% (61.1 – 61.5%)	52.1% (51.8 – 52.3%)	66.0% (65.8 – 66.2%)	
8 years rate	64.8% (64.6 – 64.9%)	55.4% (55.1 – 55.7%)	69.5% (69.3 – 69.7%)	
9 years rate	68.0% (67.8 – 68.2%)	58.7% (58.3 – 59.0%)	72.7% (72.5 – 72.9%)	
10 years rate	70.9% (70.5 – 71.2%)	61.9% (61.4 – 62.5%)	75.4% (75.0 – 75.9%)	
**MACCE – % (95% CI)**				<0.001
1 year rate	33.4% (33.3 – 33.5%)	30.6% (30.4 – 30.8%)	34.8% (34.7 – 35.0%)	
2 years rate	40.8% (40.7 – 41.0%)	35.7% (35.5 – 35.9%)	43.4% (43.2 – 43.6%)	
3 years rate	46.7% (46.6 – 46.8%)	40.0% (39.7 – 40.2%)	50.0% (49.9 – 50.2%)	
4 years rate	51.8% (51.6 – 51.9%)	43.9% (43.7 – 44.2%)	55.7% (55.5 – 55.9%)	
5 years rate	56.3% (56.2 – 56.5%)	47.7% (47.5 – 48.0%)	60.6% (60.4 – 60.8%)	
6 years rate	60.4% (60.3 – 60.6%)	51.4% (51.1 – 51.7%)	64.9% (64.8 – 65.1%)	
7 years rate	64.2% (64.0 – 64.3%)	55.0% (54.8 – 55.3%)	68.8% (68.6 – 69.0%)	
8 years rate	67.5% (67.4 – 67.7%)	58.4% (58.1 – 58.7%)	72.2% (72.0 – 72.4%)	
9 years rate	70.7% (70.5 – 70.9%)	61.7% (61.4 – 62.0%)	75.2% (75.0 – 75.6%)	
10 years rate	73.5% (73.1 – 73.8%)	64.8% (64.2 – 65.4%)	77.9% (77.5 – 78.3%)	

Abbreviation: CI, confidence interval; MACCE, major adverse cardiac and cerebrovascular events; non-ST-elevation myocardial infarction, NSTEMI; ST-elevation myocardial infarction, STEMI.
