# Supplementary material for: Differences in treatment and outcome of patients with ST- elevation myocardial infarction (STEMI) and non-STEMI in Germany
Source: PLoS One. 2026 Oct 15;21(10):e0343889. doi: 10.1371/journal.pone.0343889 (PMC13671558; doi:10.1371/journal.pone.0343889)
Supplement: Supplementary file 3 [file pone.0343889.s003.pdf]

S3 Table. Landmark analysis for death in STEMI or NSTEMI.

Endpoint – death	HR (STEMI vs NSTEMI)	95%-CI	P value
**Within 30 days**	1.84	(1.81 – 1.87)	<0.001
**Later than 30 days**	0.81	(0.80 – 0.82)	<0.001
**Within 90 days**	1.63	(1.60 – 1.65)	<0.001
**Later than 90 days**	0.80	(0.79 – 0.81)	<0.001
**Within 120 days**	1.57	(1.55 – 1.60)	<0.001
**Later than 120 days**	0.80	(0.79 – 0.81)	<0.001
**Within 180 days**	1.49	(1.47 – 1.51)	<0.001
**Later than 180 days**	0.80	(0.79 – 0.81)	<0.001
**Within one year**	1.36	(1.35 – 1.38)	<0.001
**Later than one year**	0.81	(0.79 – 0.82)	<0.001
**Within two years**	1.24	(1.22 – 1.25)	<0.001
**Later than two years**	0.82	(0.80 – 0.83)	<0.001

Abbreviations: confidence interval, CI; hazard ratio, HR; non-ST-elevation myocardial infarction, NSTEMI; ST-elevation myocardial infarction, STEMI.
