# Supplementary material for: Differences in treatment and outcome of patients with ST- elevation myocardial infarction (STEMI) and non-STEMI in Germany
Source: PLoS One. 2026 Oct 15;21(10):e0343889. doi: 10.1371/journal.pone.0343889 (PMC13671558; doi:10.1371/journal.pone.0343889)
Supplement: Supplementary file 4 [file pone.0343889.s004.pdf]

S4 Table. Prescription rates for the four essential drugs after AMI, oral anticoagulants, antiplatelet agents, statins, beta blockers and ACE-inhibitors or angiotensin receptor blockers

	STEMI	NSTEMI
**OAC – % (95% CI)**		
180 days after AMI	13.1% (12.9 – 13.3%)	22.0% (21.8 – 22.1%)
1 year after AMI	10.3% (10.2 – 10.5%)	18.1% (18.0 – 18.3%)
2 years after AMI	11.0% (10.8 – 11.2%)	19.0% (18.8 – 19.1%)
3 years after AMI	11.7% (11.5 – 11.9%)	19.8% (19.6 – 20.0%)
4 years after AMI	12.5% (12.3 – 12.8%)	20.4% (20.2 – 20.5%)
5 years after AMI	13.4% (13.1 – 13.7%)	21.2% (21.0 – 21.5%)
**PAI – % (95% CI)**		
180 days after AMI	95.9% (95.7 – 96.2%)	85.2% (85.0 – 85.4%)
1 year after AMI	91.2% (91.0 – 91.5%)	76.4% (76.2 – 76.6%)
2 years after AMI	65.0% (64.6 – 65.3%)	55.9% (55.7 – 56.2%)
3 years after AMI	60.3% (60.0 – 60.6%)	52.3% (52.1 – 52.5%)
4 years after AMI	58.0% (57.6 – 58.4%)	50.6% (50.4 – 50.9%)
5 years after AMI	56.3% (55.8 – 56.7%)	49.2% (48.9 – 49.5%)
**OAC and/or PAI – % (95% CI)**		
180 days after AMI	97.2% (97.0 – 97.4%)	91.2% (91.0 – 91.4%)
1 year after AMI	93.9% (93.6 – 94.1%)	85.1% (84.9 – 85.3%)
2 years after AMI	72.3% (72.0 – 72.6%)	69.8% (69.6 – 70.0%)
3 years after AMI	68.8% (68.5 – 69.1%)	67.6% (67.3 – 67.9%)
4 years after AMI	67.3% (66.9 – 67.7%)	66.6% (66.3 – 67.0%)
5 years after AMI	66.4% (66.0 – 66.8%)	66.2% (65.8 – 66.5%)
**Statins – % (95% CI)**		
180 days after AMI	92.6% (92.4 – 92.8%)	82.8% (82.6 – 83.0%)
1 year after AMI	85.7% (85.4 – 85.9%)	74.8% (74.5 – 75.0%)
2 years after AMI	82.7% (82.3 – 82.9%)	73.4% (73.1 – 73.6%)
3 years after AMI	81.0% (80.7 – 81.4%)	72.8% (72.5 – 73.1%)
4 years after AMI	80.2% (79.8 – 80.6%)	72.6% (72.3 – 72.9%)
5 years after AMI	79.6% (79.1 – 80.0%)	72.8% (72.5 – 73.2%)
**Beta blockers – % (95% CI)**		
180 days after AMI	92.1% (91.8 – 92.3%)	88.2% (88.0 – 88.4%)
1 year after AMI	85.0% (84.7 – 85.3%)	80.6% (80.4 – 80.8%)
2 years after AMI	82.3% (82.0 – 82.6%)	79.0% (78.7 – 79.2%)
3 years after AMI	80.9% (80.5 – 81.2%)	78.3% (78.0 – 78.6%)
4 years after AMI	80.1% (79.7 – 80.5%)	78.1% (77.8 – 78.4%)
5 years after AMI	79.8% (79.4 – 80.2%)	77.8% (77.4 – 78.2%)
**ACEI/ ARB – % (95% CI)**		
180 days after AMI	92.3% (92.0 – 92.5%)	90.1% (89.9 – 90.2%)
1 year after AMI	82.5% (82.2 – 82.7%)	79.4% (79.2 – 79.6%)
2 years after AMI	80.2% (79.9 – 80.5%)	77.8% (77.6 – 78.0%)
3 years after AMI	78.9% (78.6 – 79.3%)	77.0% (76.8 – 77.3%)
4 years after AMI	78.4% (78.0 – 78.8%)	76.7% (76.3 – 77.0%)
5 years after AMI	78.1% (77.7 – 78.5%)	76.2% (75.8 – 76.5%)

Abbreviation: AMI, acute myocardial infarction; OAC, oral anticoagulants, PAI, antiplatelet agents, ACEI, Angiotensin converting enzyme inhibitors; ARB, Angiotensin II receptor blockers
