# Supplementary material for: Differences in treatment and outcome of patients with ST- elevation myocardial infarction (STEMI) and non-STEMI in Germany
Source: PLoS One. 2026 Oct 15;21(10):e0343889. doi: 10.1371/journal.pone.0343889 (PMC13671558; doi:10.1371/journal.pone.0343889)
Supplement: Supplementary file 5 [file pone.0343889.s008.docx]

PONE-D-26-03655

Differences in Treatment and Outcome of Patients with ST- Elevation Myocardial Infarction (STEMI) and Non-STEMI in Germany

PLOS One

Journal Requirements:

When submitting your revision, we need you to address these additional requirements.

1.Please ensure that your manuscript meets PLOS ONE's style requirements, including those for file naming. The PLOS ONE style templates can be found at

https://journals.plos.org/plosone/s/file?id=wjVg/PLOSOne_formatting_sample_main_body.pdf and

<https://journals.plos.org/plosone/s/file?id=ba62/PLOSOne_formatting_sample_title_authors_affiliations.pdf>

2. We note that you have mentioned the period on which the patients were admitted to the hospital (January 1, 2010 to December 31, 2018). Please also report in the Methods section the dates when data were accessed for research purposes in DD/MM/YYYY format.

**Author’s response:** We added it the date of access for research purpose to the methods section in the main manuscript.

3. Please provide additional details regarding participant consent. In the ethics statement in the Methods and online submission information, please ensure that you have specified (1) whether consent was informed and (2) what type you obtained (for instance, written or verbal, and if verbal, how it was documented and witnessed). If your study included minors, state whether you obtained consent from parents or guardians. If the need for consent was waived by the ethics committee, please include this information.

If you are reporting a retrospective study of medical records or archived samples, please ensure that you have discussed whether all data were fully anonymized before you accessed them and/or whether the IRB or ethics committee waived the requirement for informed consent. If patients provided informed written consent to have data from their medical records used in research, please include this information.

**Author’s response:** In accordance with the decision of the Ethics Committee, no individual consent is required, as the patient data has been anonymized and it is impossible to identify individual patients based on the data provided.

4. Thank you for stating the following financial disclosure:

“The study was conducted within the framework of the GenderVasc project funded by the Federal Joint Commitee, Innovation Committee (G-BA, Innovationsfonds, number 01VSF18051)”

Please state what role the funders took in the study.  If the funders had no role, please state: 'The funders had no role in study design, data collection and analysis, decision to publish, or preparation of the manuscript.'

**Author’s response:** “The funders had no influence on the study design, data collection and analysis, the decision to publish, or the preparation of the manuscript.”

If this statement is not correct you must amend it as needed.

Please include this amended Role of Funder statement in your cover letter; we will change the online submission form on your behalf.

5. Please note that funding information should not appear in any section or other areas of your manuscript. We will only publish funding information present in the Funding Statement section of the online submission form. Please remove any funding-related text from the manuscript.

**Author’s response:** *“Funding: The study was conducted within the framework of the GenderVasc project (Gender-specific real care situation of patients with arteriosclerotic cardiovascular diseases) funded by The Federal Joint Committee, Innovation Committee (G-BA, Innovationsfonds, number 01VSF18051).”* has been removed.

If desired, we can also remove the section ***Declaration of competing interests***

*„SA Lange received support for attending meetings and / or travel by Daichi Sankyio, Medtronic, NovoNordisk and Bayer Vital, outside the submitted work. L Makowski explains the financial support for congress participation and travel expenses from Bayer Vital and Abbott, outside the submitted work. HR reports personal fees from Daiichi and StreamedUp as well as MedUpdate, DiaPlan, NeoVasc and NovoNordisk, grants from BMS/Pfizer, grants and personal fees from Pluristem, grants from Bard and Biotronik outside the submitted work. J Koeppe report research funding from the German Society for Trauma Surgery sponsored by Stryker, out-side the submitted work. C Engelbertz, P Dröge, J Gerß, C Günster and T Ruhnke have nothing to disclose.”*

6. In the online submission form, you indicated that your data is available only on request from a third party. Please note that your Data Availability Statement is currently missing the name of the third party contact or institution / contact details for the third party, such as an email address or a link to where data requests can be made. Please update your statement with the missing information.

**Author’s response:** “For assistance in obtaining access to the data, please contact [wido@wido.bv.aok.de](mailto:wido@wido.bv.aok.de).” ...is located in the “Data Availability” section on the last line.

7. Please include your full ethics statement in the ‘Methods’ section of your manuscript file. In your statement, please include the full name of the IRB or ethics committee who approved or waived your study, as well as whether or not you obtained informed written or verbal consent. If consent was waived for your study, please include this information in your statement as well.

**Author’s response:** We have implemented this change and removed the relevant items from the supplements.

8. Please remove your figures from within your manuscript file, leaving only the individual TIFF/EPS image files, uploaded separately. These will be automatically included in the reviewers’ PDF.

**Author’s response:** I hope we have done exactly what you wanted.

9. Please include captions for your Supporting Information files at the end of your manuscript, and update any in-text citations to match accordingly. Please see our Supporting Information guidelines for more information: <http://journals.plos.org/plosone/s/supporting-information>.

**Author’s response:** We have added the following to the manuscript:

**Supporting information**

**S1 Table.** ICD-10-GM Codes/ OPS diagnosis and procedure codes as well as ATC codes relevant for analyses.

**S2 Table.** Event rate including overall death, re-infarction or death and MACCE: Event rates were given by 1- survival rates determined by Kaplan Meier estimate.

**S3 Table.** Landmark analysis for death in STEMI or NSTEMI.

**S4 Table.** Prescription rates for the four essential drugs after AMI, oral anticoagulants, antiplatelet agents, statins, beta blockers and ACE-inhibitors or angiotensin receptor blockers

**S1 Fig.** Survival probability comparing STEMI vs NSTEMI over time after infarction.

**S2 Fig.** Cox regression for 90 -day survival including guideline-based medication in interaction with the type of infarction (upper panel) and including only the number of prescribed drugs (lower panel).

10. Thank you for providing your underlying data as Supporting Information.

We note that the data set contains text or data that is not in English. Please note that PLOS is an English-language publisher, so we require data sets to be provided in English as well. Please upload an English-language version of your data set.

**Author’s response:** All ‘German terms’ have been removed from the manuscript or supplemented with an equivalent English term.

This will also allow us to determine if your data follows PLOS standards per our Data Availability policy here: <https://journals.plos.org/plosone/s/data-availability>.

11. If the reviewer comments include a recommendation to cite specific previously published works, please review and evaluate these publications to determine whether they are relevant and should be cited. There is no requirement to cite these works unless the editor has indicated otherwise. 
**Author’s response:** not applicable

Additional Editor Comments:

you may want to consider eliminating the older data that covered the time of older drugs and the use of BMS in order to try to get some information that is more applicable to current day practice.

[Note: HTML markup is below. Please do not edit.]

Reviewer's Responses to Questions

**Comments to the Author**

1. Is the manuscript technically sound, and do the data support the conclusions?

The manuscript must describe a technically sound piece of scientific research with data that supports the conclusions. Experiments must have been conducted rigorously, with appropriate controls, replication, and sample sizes. The conclusions must be drawn appropriately based on the data presented.

Reviewer #1: Partly

2. Has the statistical analysis been performed appropriately and rigorously?

Reviewer #1: No

3. Have the authors made all data underlying the findings in their manuscript fully available?

The [PLOS Data policy](http://www.plosone.org/static/policies.action#sharing) requires authors to make all data underlying the findings described in their manuscript fully available without restriction, with rare exception (please refer to the Data Availability Statement in the manuscript PDF file). The data should be provided as part of the manuscript or its supporting information, or deposited to a public repository. For example, in addition to summary statistics, the data points behind means, medians and variance measures should be available. If there are restrictions on publicly sharing data—e.g. participant privacy or use of data from a third party—those must be specified.

Reviewer #1: No

4. Is the manuscript presented in an intelligible fashion and written in standard English?

PLOS ONE does not copyedit accepted manuscripts, so the language in submitted articles must be clear, correct, and unambiguous. Any typographical or grammatical errors should be corrected at revision, so please note any specific errors here.

Reviewer #1: No

5. Review Comments to the Author

Please use the space provided to explain your answers to the questions above. You may also include additional comments for the author, including concerns about dual publication, research ethics, or publication ethics. (Please upload your review as an attachment if it exceeds 20,000 characters)

Reviewer #1: In the manuscript "Differences in Treatment and Outcome of Patients with ST- Elevation Myocardial Infarction (STEMI) and Non-STEMI in Germany" the Authors address the differences in management and outcomes between STEMI and NSTEMI in Germany
The paper includes a large population and is certainly powered to assess the addressed differences
However, I have doubts with major methodological and drafting concerns and questionable statements regarding the "guidelines" indicated therapies at present days, which are not reflected in the present paper

My comments:
- The style of the text can be improved to avoid redundance (ex AMI patients with acute myocardial infarction (AMI))
- The major limitation is the period of recruitment, most likely reflecting a quite outdated cohort where most modern pharmacological and device innovations were not used. To a further confirmation almost 20% of STEMI and 10% of NSTEMI received BMS
I would suggest to account for the period of recruitment to further explain mortality data (Impella or ECMO were probably more available in most recent patients)

**Author’s response:** You’ve touched on a sensitive issue here. The cohort presented here covers the period from 2010 to 2018. Modern treatment techniques such as Impella and VA-ECMO were either not available to patients and treating physicians, or were only available at larger centers. However, we would point out that even today, VA-ECMO is only available at very large cardiology centers, such as university hospitals and cardiac centers, whilst small hospitals with cardiology departments are still permitted to provide care for heart attacks without these treatment methods. There are no regulations governing this from the health authorities in Germany.

- The Authors speak about 4 classes of guideline directed therapies. However, guidelines suggest to use RASI in patients with LV dysfunction and not systematically and for sure PAI but not OAC is indicated in AMI patients, Therefore the use of OAC should not be enclosed in the main analysis but eventually in subanalyses (probably reflecting patients with higher complexity, AF, LV thrombus etc)

**Author’s responses:**

Thank you very much for your comment. The treatment of myocardial infarction in the patients included in this study was based on the ESC guidelines on myocardial infarction from 2007 (NSTEMI) and 2008 (STEMI), as well as primarily on the 2012 guidelines. The following is quoted from the latter: Beta-blockers: *“The benefit of long-term treatment with beta-blockers following a STEMI is well established, even though these findings primarily originate from studies conducted before the advent of modern reperfusion therapies and pharmacotherapies.”*

RASI: In response to this, we would also like to refer to the old 2012 ESC guidelines, which generally recommended ACEIs or ARBs following AMI, even though it was already a matter of debate at the time and it was controversial whether all patients or only high-risk patients should receive such therapy. *“It is generally accepted that patients with a reduced ejection fraction (≤ 40%) or those in whom heart failure has developed in the early phase should be administered angiotensin-converting enzyme (ACE) inhibitors. A systematic review of studies on ACE inhibition in the early stages of STEMI found that this therapy is safe and well tolerated and is associated with a small but significant reduction in 30-day mortality, with the greatest benefit observed in the first week. Opinions continue to differ as to whether ACE inhibitors should be administered to all patients or only to high-risk patients. Patients who cannot tolerate an ACE inhibitor should receive an angiotensin receptor blocker (ARB). The use of ACE inhibitors should be considered in all patients with atherosclerosis; however, given their relatively modest effect, their long-term use cannot be regarded as strictly necessary in post-STEMI patients who are normotensive, do not have heart failure, and have neither left ventricular systolic dysfunction nor diabetes.”*

(ESC Guidelines for the management of acute myocardial infarction in patients presenting with ST-segment elevation: The Task Force on the management of ST-segment elevation acute myocardial infarction of the European Society of Cardiology (ESC) [Steg](javascript:;) PG, [James](javascript:;) SK, et al. European Heart Journal, Volume 33, Issue 20, October 2012, Pages 2569–2619, <https://doi.org/10.1093/eurheartj/ehs215>)

As it is standard practice to prescribe an anticoagulant in addition to or instead of an antiplatelet agent during further treatment, we did not explicitly distinguish between PAI and OAC in the Cox analyses. We are somewhat surprised by your objection, as this has not been questioned in our previous publications (see below), in which PAI and OAC were always analyzed together.

[Fischer AJ et al. Impact of Guideline-Directed Drug Therapy after ST-Elevation Myocardial Infarction on Outcome in Young Patients-Age and Sex-Specific Factors.](https://pubmed.ncbi.nlm.nih.gov/38999354/) J Clin Med. 2024 Jun 27;13(13):3788.

Lange SA et al., [Survival of Patients with Acute Coronary Syndrome and Hematologic Malignancies-A Real-World Analysis.](https://pubmed.ncbi.nlm.nih.gov/37894332/) Cancers (Basel). 2023 Oct 12;15(20):4966.

Engelbertz C et al. [Contemporary secondary prevention in survivors of ST-elevation myocardial infarction with and without chronic kidney disease: a retrospective analysis.](https://pubmed.ncbi.nlm.nih.gov/37915929/) Clin Kidney J. 2023 Sep 4;16(11):1947-1956.

Kuehnemund L et al., [Sex disparities in guideline-recommended therapies and outcomes after ST-elevation myocardial infarction in a contemporary nationwide cohort of patients over an eight-year period.](https://pubmed.ncbi.nlm.nih.gov/37245424/) Atherosclerosis. 2023 Jun; 375:30-37.

Engelbertz C et al., [Contemporary in-hospital and long-term prognosis of patients with acute ST-elevation myocardial infarction depending on renal function: a retrospective analysis.](https://pubmed.ncbi.nlm.nih.gov/36732721/) BMC Cardiovasc Disord. 2023 Feb 2;23(1):62.

Lange SA et al. [Acute and Long-Term Outcomes of ST-Elevation Myocardial Infarction in Cancer Patients, a 'Real World' Analysis with 175,000 Patients.](https://pubmed.ncbi.nlm.nih.gov/34944823/) Cancers (Basel). 2021 Dec 9;13(24):6203.

Unfortunately, we are no longer able to distinguish between the two groups of drugs in a Cox analysis, as contractual provisions with the relevant health insurance provider regrettably prevent us from retrieving the data again. Consequently, the requested subgroup analysis of OAC patients is not possible. However, Table S4 in the Supplements shows that 13.1% of patients with STEMI and 22% of patients with NSTEMI received an OAC within the first 180 days following the acute event, and 95.9% and 95.2% respectively received a PAI. This results in the concurrent administration of OACs and PAI within 180 days of the infarction in 9% and 7.2% of cases, respectively.

- The rate of STEMI not undergoing coronary angiography and PCI is extremely high (over 10%), as often exceeding 90% in contemporary trials.

**Author’s response:** Thank you very much for pointing this out; we had noticed it too. As our analysis reflects actual care data, this finding corresponds to the reality of AMI care outside of controlled trials in Germany.

- It is unclear if the study is retrospective or prospective. If patients were identified from insurance database, no prospective follow-up was probably performed. Otherwise, appropriate methods of patients’ follow-up (in-hospital, telephone contact, notes etc.) should be reported.

**Author’s response:** The data was collected exclusively retrospectively. In other words, past myocardial infarctions were tracked retrospectively for a period of two years prior to, and up to and including, 31 December 2019. However, the collection and analysis of the data did not take place until 2022 and was therefore carried out entirely in the past.

- As dataset was closed in 2019, it is likely that follow-up was available only for one year in some patients. A flow-chart including patients excluded- lost to follow-up and study design would help to clarify many aspects

**Author’s response:** The dataset was closed 31/12/2019. We no longer have access to data from 2020 onwards. A retrospective analysis of patients who could be followed up for less than two years is no longer possible due to the contractual restrictions mentioned earlier.

- Statistical analysis for baseline differences is not provided, nevertheless Authors state that there was a difference between STEMI and NSTEMI. Was it only numerical? Where are the tests described in Supplementary methods? To this reviewer the Statistical methods should be completely described in the main paper

**Author’s response:** The statistical methods were described in detail in the supplements. As requested, we are including the detailed statistical section in the main manuscript.

- What variables were included in the multivariable model? Which tests were used in them? Were they different between NSTEMI and STEMI?

**Author’s response:** Multivariable Cox regression analysis with time-dependent covariables were used to evaluate the association of infarction type on overall and long-term survival and to evaluate the treatment effect of different guideline-rated drug therapies. In a first step, time-dependent risk association of the infarction type was analyzed using different models. First, overall survival was modeled using a time-dependent variable (STEMI vs NSTEMI). Second, a landmark analysis was performed. To address differences between the associations of guideline-directed medication beta blockers (ATC: C07), ACEI/ARBs (ATC C09A-D), statins (ATC: C10AA, C10BA, C10BX) and PAI (ATC: B01AC) or OAC (ATC: B01AA, B01AE07, B01AF (from 2013), B01AX06 (only 2012), B01AX08 (only 2012) on overall and long-term survival between patients with STEMI or NSTEMI, an interaction term of infarction type (STEMI vs NSTEMI) and pharmaceutical therapy was added to the models. Model fit was performed in two ways: First, all five drugs were considered individually and, second, only the number of prescribed drugs were considered. The rate of prescribed medication during follow-up was evaluated by determining the actual state probabilities of the respective multi-state models using Aalen-Johansen estimates.

- Which drugs were more likely discontinued? Most recent evidence shows no persistent benefit by continuing blockers. This should be discussed and data analyzed for this specific endpoint

**Author’s response:** Table S4 in the Supplements provides a detailed summary of which drugs were no longer prescribed following STEMI and NSTEMI. Over a five-year period, the prescription rate for statins following STEMI fell from an initial 92.6% to 79.6%, and for NSTEMI from 82.8% to 72.8%. For beta-blockers, the prescription rate following STEMI fell from 92.1% to 79.8% and for NSTEMI from 88.2% to 77.8%. For ACEIs/ARBs, the initial prescription rate for STEMI was 92.3% and fell to 78.1%; for NSTEMI, the trend was similar, falling from an initial 90.1% to 76.2%.

Platelet inhibition decreased from an initial 95.9% to 66.4% in STEMI patients. In NSTEMI patients, this rate fell from 85.2% to 49.2%.

The prescribing situation is different for OACs. Thus, 13.1% of STEMI patients received an OAC within the first 180 days and 13.4% were still receiving an OAC after 5 years. Among NSTEMI patients, as many as 22.0% received an OAC at the outset and 21.2% after 5 years.

The benefits of beta-blocker therapy in AMI patients with reduced ejection fraction (<40%) are undisputed. However, there was no clear evidence of sustained benefit following STEMI or NSTEMI in patients with preserved ejection fraction. Nevertheless, older pooled data have shown that STEMI patients experienced a reduction in mortality of approximately 20% (ESC ACS Guideline 2023). Even though our data originate from an era based on reperfusion following AMI, our analyses yield comparable results. Thus, the median mortality is reduced with an **HR of 0.85 [CI 0.81, 0.89] for STEMI and 0.93[CI 0.90, 0.95]** for NSTEMI. Our study did not distinguish between patients with preserved or reduced LV function. There was no difference in prescribing patterns between the main agents following AMI.

As requested, we have added the following contribution to the discussion:

The use of beta-blockers following an acute myocardial infarction with reduced left ventricular function has long been undisputed [1, 27]. By contrast, the general use of beta-blockers following an AMI – regardless of left ventricular function – has long been a matter of debate [1]. Recently published studies, summarized in two meta-analyses, fills this gap in knowledge. In patients with AMI and a left ventricular ejection fraction (LV-EF) of at least 50%, beta-blockers had no effect on overall mortality [31]. In contrast, in patients with mildly impaired cardiac function (LV-EF 40–49%), beta-blocker therapy provides a survival benefit [32]. However, our analysis—which grouped all STEMI and NSTEMI patients together regardless of concomitant heart failure—revealed a similar reduction in all-cause mortality among patients taking beta-blockers, consistent with the findings of the nationwide Korean cohort study on long-term beta-blocker therapy following AMI [33].

We added following references:

**31.** Kristensen AMD, Rossello X, Atar D, Yndigegn T, Kimura T, Latini R, Lindahl B, Halvorsen S, Olsen MH, Fuster V, Hofmann R, Vikenes K, Maeng M, Erlinge D, Pocock S, Karlström P, Bakken A, Lange T, Barrabés JA, Benatar J, Raposeiras-Roubin S, Held C, Piepoli M, Fagerland MW, Holmager T, Ozasa N, Prescott EIB, Munkhaugen J, Jernberg T, Ibanez B; Beta-Blocker Trialists’ Collaboration Study Group. [Beta-Blockers after Myocardial Infarction with Normal Ejection Fraction.](https://pubmed.ncbi.nlm.nih.gov/41211954/) N Engl J Med. 2026 Feb 5;394(6):540-550. doi: 10.1056/NEJMoa2512686. Epub 2025 Nov 9. PMID: 41211954

**32.** Rossello X, Prescott EIB, Kristensen AMD, Latini R, Fuster V, Fagerland MW, Pocock SJ, Halvorsen S, Dominguez-Rodriguez A, Holmager TLF, Sanchez PL, Bakken A, Raposeiras-Roubin S, Jensen SE, Kimura T, Ottani F, Lambrechtsen J, Anguita M, Ozasa N, Atar D, Ibanez B, Munkhaugen J. [Beta blockers after myocardial infarction with mildly reduced ejection fraction: an individual patient data meta-analysis of randomised controlled trials.](https://pubmed.ncbi.nlm.nih.gov/40897190/) Lancet. 2025 Sep 13;406(10508):1128-1137. doi: 10.1016/S0140-6736(25)01592-2. Epub 2025 Aug 30.PMID: 40897190

**33.** Kim J, Kang D, Park H, Kang M, Park TK, Lee JM, et al. Long-term β-blocker therapy and clinical outcomes after acute myocardial infarction in patients without heart failure: nationwide cohort study. Eur Heart J 2020;41:3521–3529. https://doi.org/10.1093/ eurheartj/ehaa376

- More modern agents, as newer lipid acting agents, were not analyzed, and neither target LDL (which is more important in guidelines as compared to therapy)

**Author’s response:** This is correct. With the data made available to us, it was and remains impossible to retrieve treatment outcomes such as patients’ cholesterol or LDL levels, as these are not recorded by health insurance providers. Newer forms of lipid-lowering therapy were not taken into account, as – with the exception of Ezetrol – they were not available for general patient care prior to 2019. Nevertheless, we would point out that statin therapy remains the cornerstone of cholesterol-lowering treatment even today, and that PCSK-9 inhibitors and/or bembedoic acid are usually only added when there is statin intolerance or when cholesterol targets are not met.

- Figures: KM curves are low quality, if landmark analysis was performed at 3 months this should be better highlighted

**Author’s response:** If you prefer, we can provide Figure S1 from the supplements instead of Figure 1. This figure is based on the same data, and the timeline is clearer here.

6. PLOS authors have the option to publish the peer review history of their article ([what does this mean?](https://journals.plos.org/plosone/s/editorial-and-peer-review-process#loc-peer-review-history)). If published, this will include your full peer review and any attached files.

If you choose “no”, your identity will remain anonymous but your review may still be made public.

**Do you want your identity to be public for this peer review?** For information about this choice, including consent withdrawal, please see our [Privacy Policy](https://www.plos.org/privacy-policy).

Reviewer #1: **Yes:** Monica Verdoia

[NOTE: If reviewer comments were submitted as an attachment file, they will be attached to this email and accessible via the submission site. Please log into your account, locate the manuscript record, and check for the action link "View Attachments". If this link does not appear, there are no attachment files.]

To ensure your figures meet our technical requirements, please review our figure guidelines: https://journals.plos.org/plosone/s/figures

You may also use PLOS’s free figure tool, NAAS, to help you prepare publication quality figures: https://journals.plos.org/plosone/s/figures#loc-tools-for-figure-preparation.

NAAS will assess whether your figures meet our technical requirements by comparing each figure against our figure specifications.
