# Supplementary material for: Differences in treatment and outcome of patients with ST- elevation myocardial infarction (STEMI) and non-STEMI in Germany
Source: PLoS One. 2026 Oct 15;21(10):e0343889. doi: 10.1371/journal.pone.0343889 (PMC13671558; doi:10.1371/journal.pone.0343889)
Supplement: Supplementary file 6 [file pone.0343889.s009.docx]

"Reviewer #2: This is a large, contemporary, real-world study with impressive statistical power (>540,000 patients) that compared prognosis of STEMI and NSTEMI patients and evaluated prescription of guideline-directed medical therapy (GDMT) and survival in these patients. All parts of the manuscript are well written: the introduction gives important information and current literature and guidelines overwiev. Methodology is well written; authors precisely explained patients' selection. Results are clearly presented in tables and figures. Discussion is well conducted and conclusions are in concordance with findings. Cited literature is relevant and is up-to-date.
The authors correctly note that NSTEMI represents a more heterogeneous population. In the presented results, NSTEMI patients appear older and have a higher burden of comorbidities including CKD, PAD, and heart failure. Therefore, comparisons of long-term outcomes between STEMI and NSTEMI may primarily reflect differences in baseline risk rather than differences attributable to infarct type itself.
***I would suggest that authors clearly describe all variables included in multivariable adjustment and consider propensity score matching. Present adjusted survival curves.***
Thank you"

Answer to the reviewer # 2:

In the supplements we described in detail the statistical analysis. For your question you can find the answer in this part:

*“The Kaplan-Meier estimates were determined for overall survival stratified by sex and age and differences between STEMI and NSTEMI patients were tested using two-sided log-rank test for all patients and respectively in each age group (<40 years, 40-49 years, 50-59 years, 60-69 years, 70-79 years, 80- 89 years, ≥90 years). Multivariable logistic regression model (30-days mortality) and multivariable Cox regression models with time-dependent co-variables (OS, MACE, long-term survival, re-infarction) were performed to evaluate the association between sex and short- and long-term outcomes adjusted by patient’s risk profile. To evaluate the association of guideline recommended medications at discharge, model fit was performed in two ways: First, all four drugs were considered individually and, second, only the number of prescribed drugs were considered. In the models, prescriptions within 90 days after discharge were included as time-dependent factors. Thereafter, the respective variable remained constant over the entire follow-up. In order to address different patient’s risk profiles between STEMI and NSTEMI patients for 30-day mortality and OS, the interaction of sex with all other variables in the respective models were evaluated. Especially, differences between STEMI and NSTEMI patients were evaluated in this way for the association of the pharmacotherapy recommended by the guideline in discharge to the OS. P-values of the Odds ratio (OR) comparing STEMI and NSTEMI patients of the logistic regression model. Hazard ratios (HR’s) comparing STEMI and NSTEMI patients of all Cox regression models. The p-values for the test of interaction of all models and all p-values comparing Kaplan-Meier estimates for STEMI and NSTEMI patients were jointly adjusted using Benjamini-Hochberg procedure [37] to control the false discovery rate (FDR) with respect to the multiple comparison problem.”*

Thank you very much for your advice to include propensity score matching in this work. I would like to respond as follows: We have already performed a statistical analysis for STEMI and NSTEMI patients, using a two-sided log-rank test for all patients, as well as in each age group, and a multivariable logistic regression model, as well as multivariable Cox regression models with time-dependent covariates, to evaluate the association between sex and short- and long-term outcomes, adjusted by the patient’s risk profile. An additional propensity score match does not seem necessary, and unfortunately this is no longer possible due to restricted data access from the healthcare provider.
